# Exploring the Perspective of Oral Microbiome Studies in PubMed Database: A Bibliometric Appraisal

**DOI:** 10.7759/cureus.53824

**Published:** 2024-02-08

**Authors:** Namrata Dagli, Susmita Sinha, Mainul Haque, Santosh Kumar

**Affiliations:** 1 Dentistry, Karnavati Scientific Research Center (KSRC) Karnavati School of Dentistry, Karnavati University, Gandhinagar, IND; 2 Physiology, Khulna City Medical College and Hospital, Khulna, BGD; 3 Pharmacology and Therapeutics, National Defence University of Malaysia, Kuala Lumpur, MYS; 4 Periodontology and Implantology, Karnavati Scientific Research Center (KSRC) Karnavati School of Dentistry, Karnavati University, Gandhinagar, IND

**Keywords:** microflora, potent modulator of pro-inflammatory, factorial analysis, thematic analysis, network analysis, bibliometric analysis, pubmed database, oral microbiota, oral microbiome, microbioma

## Abstract

This research aims to postulate an exhaustive sketch of the current research background on the oral microbiome to emphasize prevailing research trends. On November 25, 2023, a digital exploration was conducted on the PubMed platform. The search strategy employed was- ‘(Microbiome, Microbiota, Microorganisms, Bacteria, Virus, Fungi) AND (Oral, Dental, Saliva, Plaque, Gingival Crevicular Fluid)'. Inclusive criteria comprised review articles, clinical trials, and meta-analyses. The Biblioshiny app and VOSviewer software were used to create and visualize bibliometric maps for network, thematic, and factorial analyses.

The PubMed database search unveiled 215,068 published research studies on the oral microbiome, indicating a fluctuating publication pattern with an all-embracing mounting trajectory. Notably, there was a substantial increase in publications in 2020 and 2021, succeeded by a marked decline in 2022 and 2023. Sichuan University and the International Journal of Molecular Sciences emerged as the most prolific contributors among organizations and relevant sources. Keyword analysis revealed a research emphasis on the COVID-19 pandemic and the SARS-CoV-2 virus since 2019. Thematic mapping categorized key terms into motor, primary, niche, and emerging themes. The emerging terms identified are viral immunogenicity, antibodies, and vaccines, which support the revelation that COVID-19 and related terms will be the most pertinent subjects in oral microbiome studies in the future. Factorial analysis delineated the relationships between topics and subtopics in this domain.

## Introduction and background

The human oral cavity harbors a dynamic and complex ecosystem known as the oral microbiome, which encompasses a vast array of microorganisms, including bacteria, viruses, fungi, and archaea. The term was coined by Joshua Lederberg in 2001 [1]. The intricate interplay within this microbial community significantly influences not only oral health but also systemic well-being. The oral microbiome plays a pivotal role in various physiological processes, ranging from digestion to immune modulation. At the same time, dysbiosis in this microbial community has been implicated in the pathogenesis of numerous oral and systemic diseases [2-4].

According to the study done by Jiang W et al., 2024, the composition of oral bacteria exhibits specificity during various stages of the progression of dental caries [5]. Furthermore, established connections have been identified between oral dysbiosis and cardiovascular diseases (CVDs), encompassing conditions such as atherosclerotic diseases, heart failure, infective endocarditis, and rheumatic heart disease, which might involve several mechanisms, including immunomodulation, endothelial dysfunction, antibody cross-reactivity, protein citrullination, arterial invasion, platelet activation, aggregation and thrombogenesis [6]. The microbiome has recently been recognized as a factor contributing to differences between individuals, marked by person-specific characteristics. Consequently, the microbiome has the potential to influence the development of diseases, even in individuals who share similar genetic susceptibility risks [7]. Thus, Ratiner et al., 2023 altering the microbiome profile might represent a more personalized, safe, and effective treatment strategy for individuals [8].

Over the years, research on the oral microbiome has witnessed a burgeoning interest, driven by advancements in high-throughput sequencing technologies, metagenomics, and bioinformatics. As the field of oral microbiome research continues to evolve, it becomes imperative to conduct a bibliometric analysis to systematically evaluate the trends, patterns, and key contributors shaping this scientific landscape. Bibliometric analyses quantitatively assess collaboration networks, thematic evolution, and research trends within a research domain.

This paper aims to provide an overview of the current state of oral microbiome research through a comprehensive bibliometric analysis, shedding light on key research themes, influential publications, collaborative networks, emerging trends, landmark studies, most productive authors, and impactful journals. Additionally, it aims to uncover collaborative networks among researchers and institutions, facilitating a deeper comprehension of the global landscape of oral microbiome research. The findings from this bibliometric analysis are expected to not only contribute to the existing body of knowledge in oral microbiome research but also inform future directions for scientific inquiry and collaboration in this rapidly expanding field.

## Review

Materials and methods

An internet search was accomplished on 25 November 2023 in the PubMed database to explore the research done on oral microbiomes. The search strategy employed was- '(Microbiome, Microbiota Microorganisms, Bacteria Virus, Fungi) AND (Oral, Dental, Saliva, Plaque, Gingival Crevicular Fluid).' We arranged the published studies chronologically and exported data in a text file for further analysis. Due to limitations in the PubMed database, we could export data from only 10,000 studies published between 2019 and 2023. Clinical trials and reviews, including systemic reviews and meta-analyses, were included without filtering for species, linguistics, sex, journal, era, or publication time. The step-by-step diagram of the study collection procedure was initiated, conferring to the PRISMA guidelines [9].

Subsequently, the data was exported to text files for subsequent analysis. Network analysis and visualization were done with the help of the VOSviewer software (version 1.6.19) and the Biblioshiny App [15]. VOSviewer is a software tool for creating and visualizing maps based on network data [10]. Biblioshiny is a web-based app for wide-ranging science representing exploration [11].

The following features were evaluated: "i. Research trends, ii. Most frequently used keywords, iii. Leading organizations, iv. Most contributing countries, v. Most relevant journals, vi. Co-occurrences and link strengths among various keywords, vii. Trend topic, thematic, and factorial analysis related to oral microbiome research" [9].

Results

**Figure 1:**
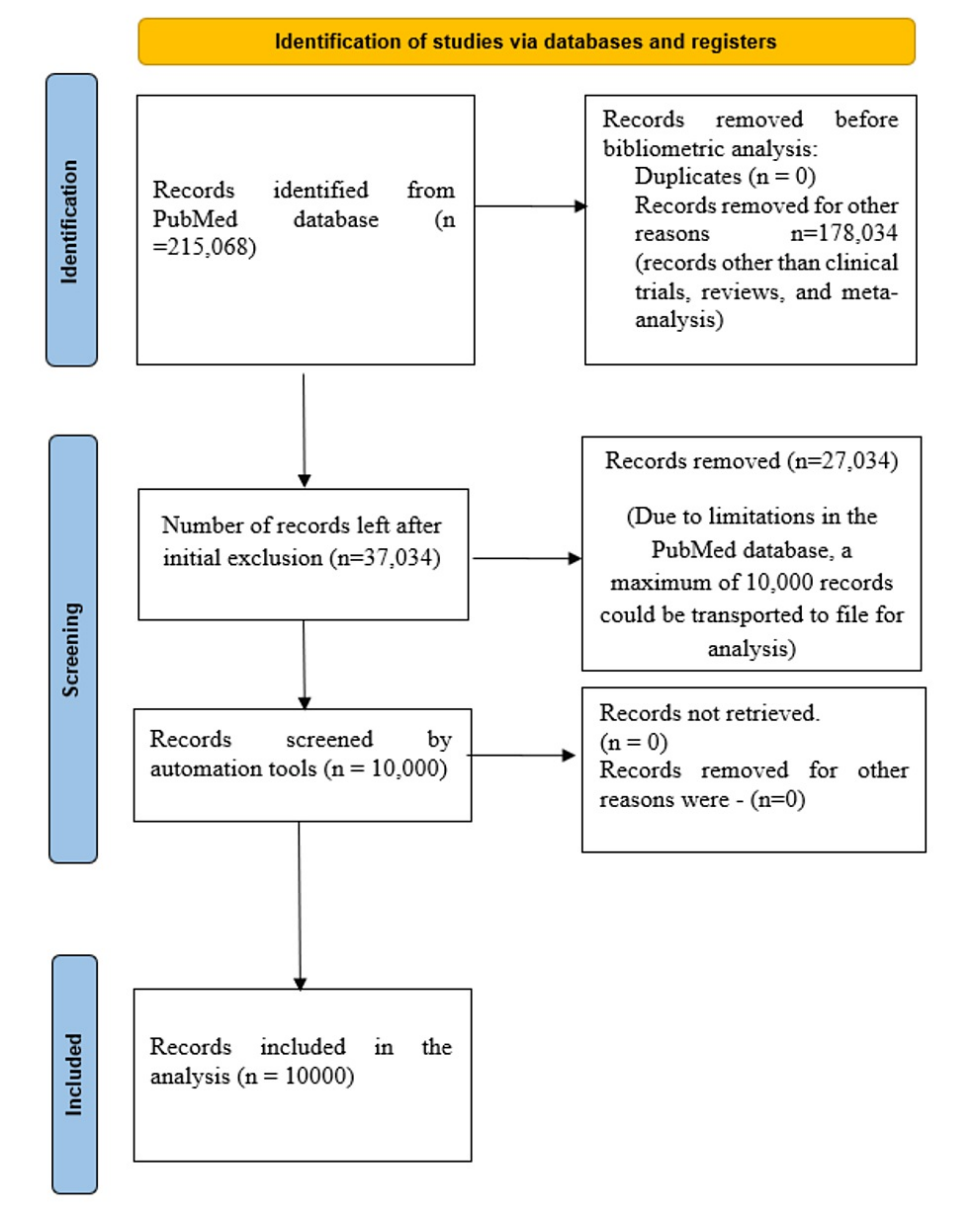
A schema chart depicting the process of selection of studies

An internet-based exploration in the PubMed repository yielded a total of 215,068 results. Among these were 11,374 clinical trials, 24,734 reviews and systematic reviews, and 926 meta-analyses. All the articles falling under these categories were considered, while all other types of documents (n=178,034) were excluded (Figure 1). Due to a limitation in the PubMed database, only the most recent 10,000 articles could be exported in a text file, and thus, this analysis focused on that subset.

Publishing Trend of Research Papers on Oral Microbiome

**Figure 2:**
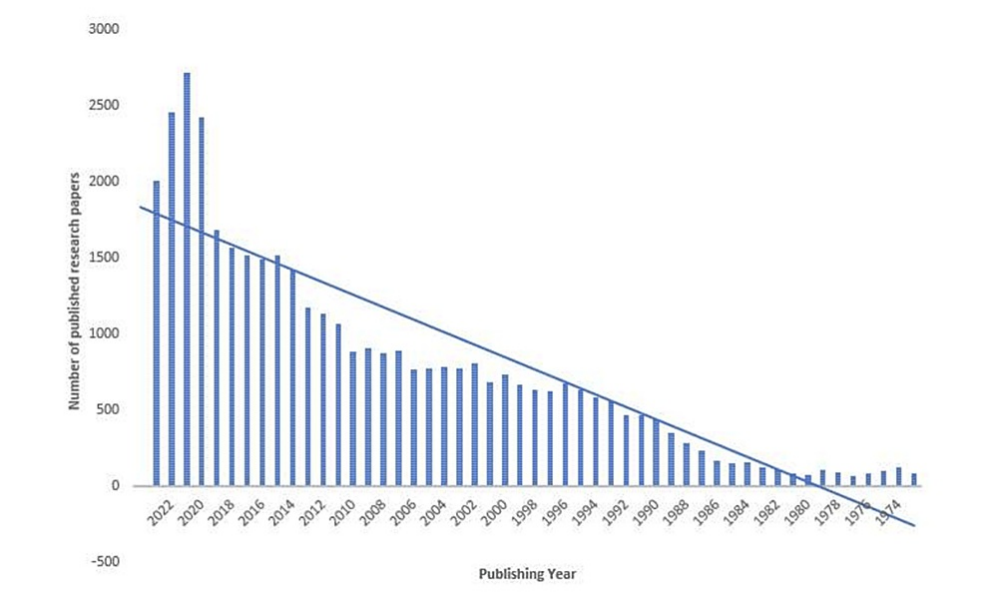
The publishing trend of research papers on oral microbiome

The PubMed database search revealed 215,068 published documents on oral microbiomes, displaying an irregular pattern in the number of publications. Despite this irregularity, the trend line indicates an overall increase. Notably, a significant surge is observed in 2020 and 2021, followed by a substantial decline in 2022 and 2023 (Figure 2).

Most Relevant Affiliations

**Figure 3:**
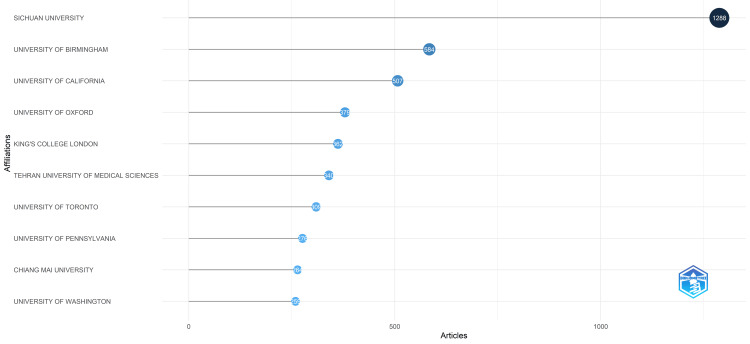
Most Relevant Universities in the Oral Microbiome Research

Sichuan University emerged as the foremost institution in oral microbiome research based on the number of published studies on oral microbiomes, boasting 1288 publications. This university constitutes over a quarter (28.1%) of the combined output from the ten most relevant universities or institutions identified in our analysis by Biblioshiny software. It is a Java software designed and constructed by Professor Massimo Aria in Statistics for Social Sciences at the Department of Economics and Statistics of the University of Naples Federico, Italy [12-14]. The total number of papers published by all ten most relevant universities/institutions is 4568. Notably, the University of Birmingham and the University of California have also made significant contributions, publishing 584 and 507 research papers, respectively (Figure 3).

Most Relevant Sources

**Figure 4:**
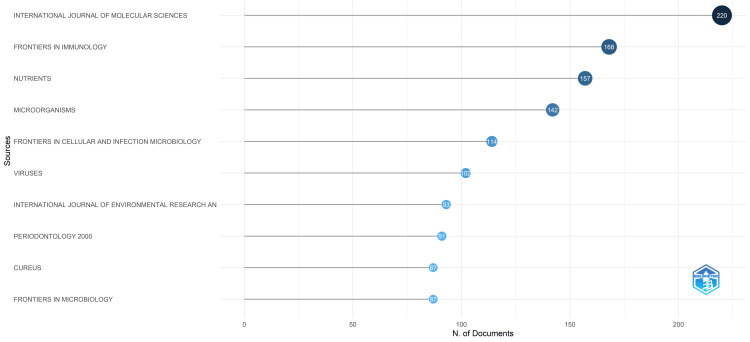
Most Relevant Sources in the Oral Microbiome Research

The International Journal of Molecular Sciences emerged as the most relevant source based on the number of studies published on oral microbiomes. It published 220 research papers on oral microbiomes during the last five years. Notably, this journal accounts for nearly 17.45% of the documents published by the ten most relevant journals identified in our analysis by Biblioshiny software [12-14]. The total number of papers published by all ten most relevant journals is 1261. Other significant contributors include Frontiers in Immunology, Nutrients, and Microorganism, each presenting 168, 157, and 142 research papers, respectively (Figure 4).

Keywords Analysis

**Figure 5:**
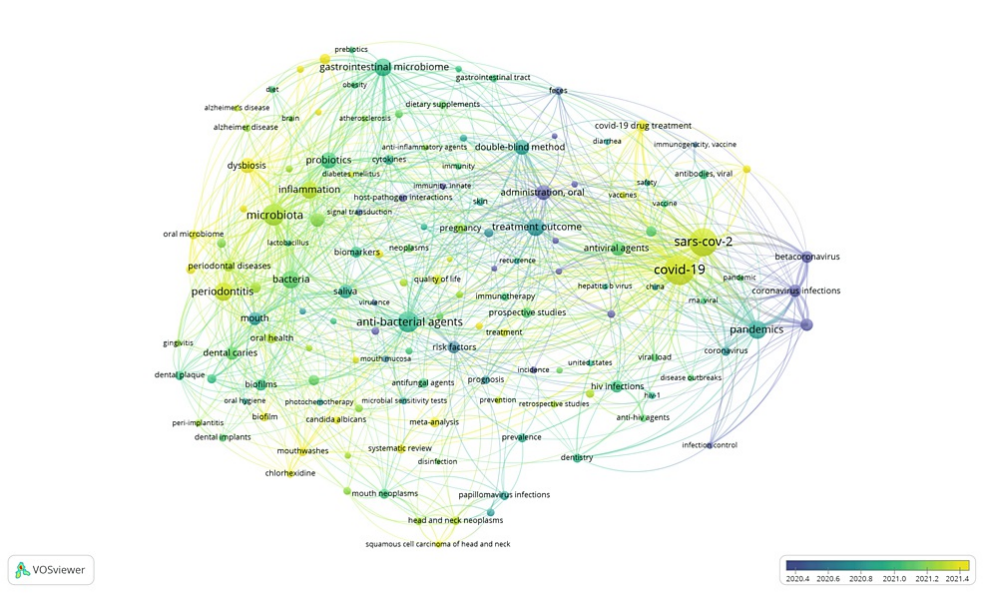
Overlay visualization of co-occurrence of keywords in research papers on oral microbiome Notes: The colors of nodes and links represent the publishing year (Weight: Occurrences, Scores: Average publication, Labels’ maximum length: 50)

The bibliometric analysis of the 10,000 most recent documents on the oral microbiome revealed a total of 23,545 keywords. Among these, 3,024 keywords repeated at least five times, and 158 had a minimum occurrence of 75 times. The overlay visualization incorporated 142 interconnected keywords, forming 5 clusters with 6,144 links and a combined link strength of 47,062. The clusters varied in size, ranging from 11 to 38 items. To enhance clarity, specific keywords unrelated to the oral microbiome were excluded from the overlay visualization, such as human, female, adult, child, male, animal, humans, animals, middle-aged, infants, mice, double-anonymized method, aged, adolescent, infant, young adult, children, child, and preschool (Figure 5). An overlay visualization of the co-occurrence of keywords used in research papers summarizes how different terms are connected and how frequently they appear together in the scientific literature.

**Figure 6:**
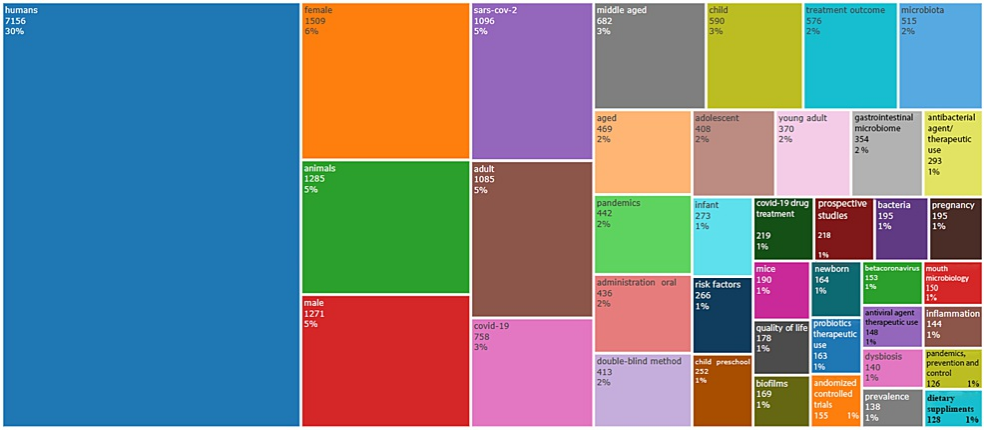
Treemap of keywords in Research papers on oral microbiome

Figure 6 presents a tree map illustrating the most frequently used keywords and their frequencies. All the keywords were included in the Treemap. Notably, the keyword analysis suggests a greater emphasis on research related to COVID-19 and the SARS-Cov-2 virus in humans, particularly adult females.

**Figure 7:**
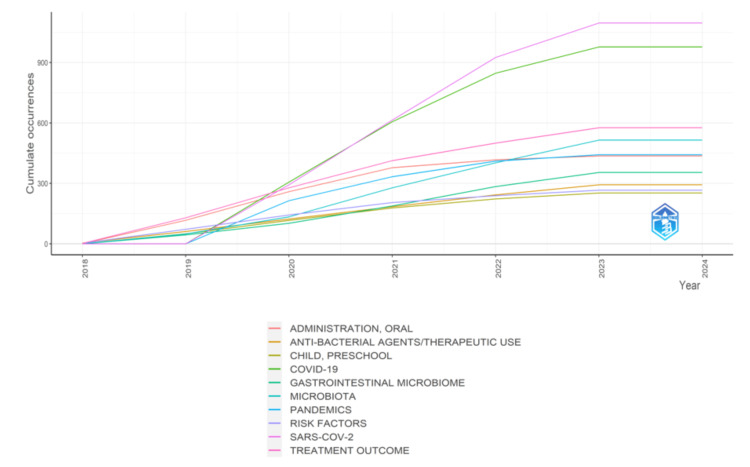
Keywords’ frequency over time in Research papers on oral microbiome

Figure 7 represents the frequency of the keywords: Oral administration, antibacterial agents/ therapeutic use, preschool child, COVID-19, gastrointestinal microbiome, microbiota, pandemics, risk factors, SARS-COV-2, and treatment outcome. The graph suggests that the research has been focused on the COVID-19 pandemic and the SARS-COV-2 Virus since 2019.

Trend Topic Analysis

**Figure 8:**
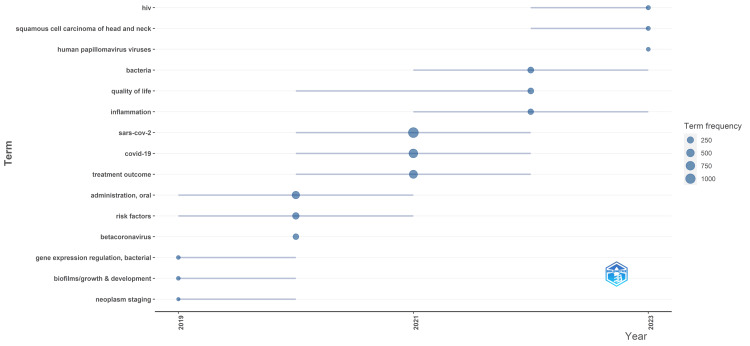
Trend topic analysis of Research papers on oral microbiome

The analysis of topic trends indicates that in 2019, oral microbiome research predominantly centered around bacteria, gene expression regulation, biofilms, and neoplasm staging. However, from 2020 to 2022, different themes emerged, including betacoronavirus, oral administration, treatment outcomes, COVID-19, SARS-CoV-2, inflammation, and quality of life. The most recent terms identified are HIV, Human Papilloma Virus, and squamous cell carcinoma of the head and neck. This suggests a shift in research focus from bacterial biofilms towards viruses, particularly Sars-COV-2, HIV, and Human Papilloma Virus (Figure 8).

To refine the specificity of words related to oral microbiome research, the topic trend analysis excluded specific nonspecific keywords such as human, female, adult, child, male, animal, humans, animals, middle-aged, infants, mice, double-anonymized method, aged, adolescent, infant, young adult, and child preschool. Additionally, synonyms were consolidated to enhance the accuracy of the results.

Thematic Map and Thematic Evolution

**Figure 9:**
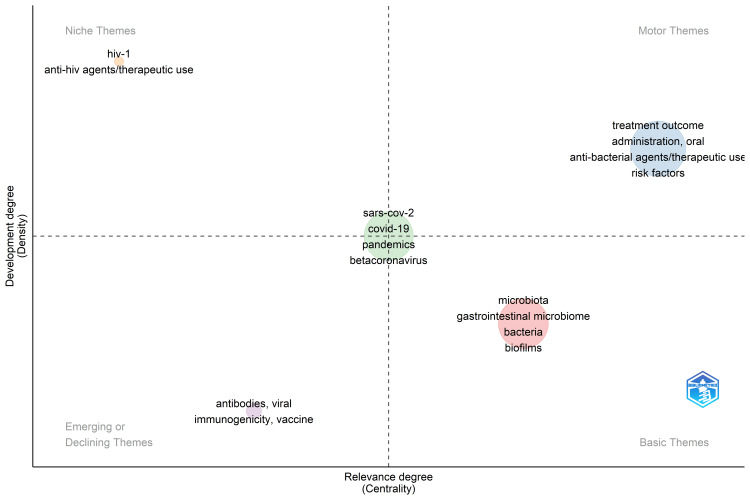
Thematic Map Notes: The size of the circle reflects the frequency of the themes used in research papers on oral microbiome

An ideological portrait serves as a visual representation delineating the thematic structure of the research field. It categorizes themes into motor, basic, transversal, highly developed, isolated, and emerging or declining themes. The themes that guide the field are motor themes, including treatment outcomes, antibacterial agents, oral administration, therapeutic uses, and risk factors. Niche themes, though not extensively interconnected, bear significant importance, including HIV-1, anti-HIV agents, and medicinal uses. Emerging themes that are gaining prominence include viral, antibodies, vaccines, and immunogenicity. Basic themes firmly established within the field cover microbiota, gastrointestinal microbiome, bacteria, and biofilms. The thematic map indicates that COVID-19, SARS-CoV-2, Beta coronavirus, and the pandemic are the most relevant themes in research on the oral microbiome (Figure 9). To enhance the specificity of words in the thematic map and thematic evolution analysis, nonspecific keywords were excluded, as mentioned in the topic trend analysis.

**Figure 10:**
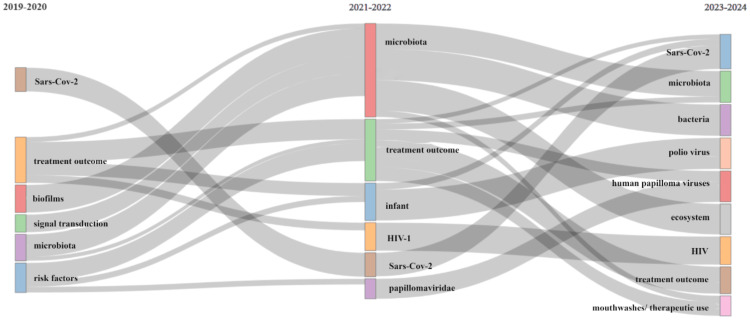
Thematic Evolution analysis (by Biblioshiny)

Analysis of thematic evolution (Figure 10) reveals that the terminology employed over the past five years has remained broadly consistent. Standard terms, including SARS-CoV-2, treatment outcome, microbiota, biofilms, and HIV, persist, indicating a consistent research focus during this period.

Factorial Analysis

**Figure 11:**
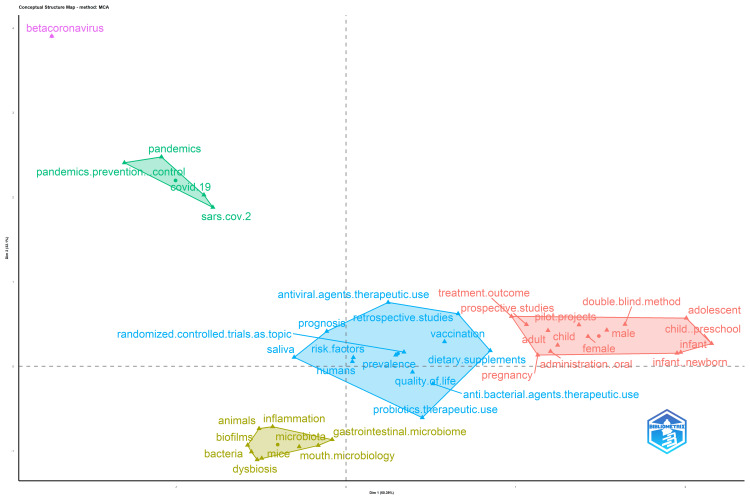
Factorial Map

The conceptual structure map (Figure 11) produced through multiple correspondence analysis reveals 45 terms organized into 5 clusters. No distinct relationships are discernible. The smallest cluster comprises only one keyword, "betacoronavirus." The largest cluster, represented in blue, showcases vital terms employed in the oral microbiome research papers, indicating the types and themes of included studies. These encompass retrospective studies, randomized controlled trials, saliva, vaccination, probiotics, antibacterial agents, antiviral agents, dietary supplements, prevalence, and quality of life.

Keywords within the pink clusters predominantly pertain to the characteristics of the study population, including descriptors such as male, female, adult, child, infant, and adolescent. This suggests that the study population encompasses individuals across all age groups. The green cluster's keywords are linked to the COVID-19 pandemic. In contrast, those in the yellow cluster are associated with the microbiome, encompassing terms like biofilms, bacteria, mouth microbiology, gastrointestinal microbiome, and dysbiosis. To enhance accuracy, synonyms were consolidated during the generation of the factorial map.

Topic Dendrogram

A topic dendrogram is a visual representation that arranges topics in a hierarchical structure, elucidating their relationships based on content similarities. The branching patterns demonstrate how topics undergo grouping and subdivision as the algorithm identifies similarities. Closer-related topics form clusters at lower dendrogram levels, whereas more distant or dissimilar topics are positioned at higher levels.

**Figure 12:**
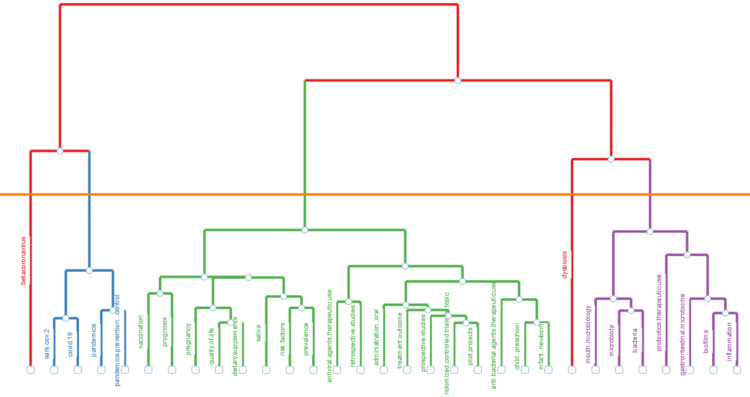
Topic dendrogram

In Figure 12, the dendrogram specifically denotes research topics correlated with oral microbiome research. Various colors are employed to emphasize distinct clusters or themes. Keywords sharing the same color are closely associated with one another. To enhance the precision of words pertinent to oral microbiome research, the dendrogram excludes specific nonspecific keywords, such as human, female, adult, child, male, animal, humans, animals, middle-aged, infants, mice, double-blinded method, aged, adolescent, infant, young adult, and child preschool.

Discussion

The PubMed database search unveiled 215,068 published documents on the oral microbiome, exhibiting a fluctuating publication pattern; however, the trend line reveals a generally upward trajectory, with a prominent surge in 2020 and 2021 followed by a substantial decline in 2022 and 2023. The analysis identified Sichuan University as the most productive organization and the International Journal of Molecular Sciences as the most relevant source in this field.

The bibliometric analysis of the 10,000 most recent documents on the oral microbiome revealed a total of 23,545 keywords, the study of which suggests a greater emphasis on research related to COVID-19 and the SARS-CoV-2 virus in humans, particularly adult females. Thematic map displays the spatial distribution of a specific theme related to oral microbiome and categorizes them into- motor, essential, niche, and emerging themes. Also, it highlighted COVID-19 and related terms as the most prominent themes in the oral microbiome research. The thematic evolution analysis in our study suggests that the research community has maintained its attention on topics related to SARS-CoV-2, treatment outcomes, microbiota, and HIV over the last five years, as evidenced by the persistent usage of these terms in the analyzed literature or data. The conceptual structure map generated through multiple correspondence analysis reveals 45 terms organized into 5 clusters, highlighting key terms related to the oral microbiome research. The topic dendrogram (Figure 12) visually represents hierarchical relationships among research topics in oral microbiome research. However, other bibliometric studies on the microbiome's relationship with gastric cancer, COPD, and other diseases have been found [15-18], and bibliometric studies addressing oral microbiome research are very scarce.

A recent study conducted by Li et al. in 2023, utilizing the Web of Sciences, pinpointed the United States and China as the principal contributors to oral microbiome research, with Forsyth Institution leading the organizations and Sichuan University and Frontiers in Cellular and Infection Microbiology as noteworthy sources. The analysis also revealed a shift from treatment to prevention. However, comparing these findings with our study is not feasible due to variations in the data sources [19]. Another bibliometric study by Liao et al., 2020 analyzed data from Thomson Reuters' Web of Science Core Collection until December 31st, 2019, identifying 2225 articles and reviews. The United States and Harvard University emerged as the top countries and institutions, while keywords analysis revealed periodontal disease, oral microbes, and dental plaque as research focal points [20]. Nonetheless, differences in our study's findings are attributed to disparities in the databases used and potentially to variations in the selected periods for analysis [21].

It is very important to understand the bacterial spectrum in health and disease. Numerous recent studies have sought to delineate the microbiome in various pathological conditions. A study by Yankov YG et al. reported bacterial species isolated from odontogenic and nonodontogenic abscesses. Gram-positive bacteria isolated from odontogenic abscess were coagulase-negative *Staphylococci*, *Staphylococcus aureus*, *Streptococcus anginosus*, and *Streptococcus viridans*. Gram-negative bacteria isolated from odontogenic abscesses were *Escherichia coli*, *Enterobacter cloacae*, and Stenotrophomonas maltophilia. Obligate anaerobes were found in only one sample. The isolated fungi were Candida albicans and Candida nonalbicans [22]. Another study identified *S. aureus*, *C. freundii*, *E. coli*, *S. pyogenes*, and *K. oxytoca* represented by 3 each of all isolates. *P. aeruginosa*, *S. pneumoniae*, *E. faecalis*, *Prevotella oris*, and *Citrobacter *​​​​​​*diversus* and *Candida Albicans* [23,24]. A comprehensive understanding of the lymph nodes of the head and neck is essential for making the correct diagnosis and determining the treatment plan [25]. The isolated microorganisms from neck abscesses of lymph node origin were of the gram-positive spectrum - *Staphylococcus aureus*, *Staphylococcus hemolyticus*, *Staphylococcus epidermidis*, and *beta-hemolytic streptococci.* Gram-negative bacteria were - *Klebsiella pneumoniae*, *Bartonella henselae*, *Klebsiella oxytoca*, *Enterobacter cloacae,* and *Flavimonas oryzihabitans*. More than one bacterial species represents gram-positive resident microflora. No anaerobic and fungal microorganisms were isolated [26]. Additionally, other studies reported bacteria associated with sialoadenitis and phlegmosis of the mouth floor [26,27]. More studies are required to stay abreast of evolving microbial landscapes, address antibiotic resistance, improve diagnostics, understand systemic implications, optimize treatments, implement preventive measures, and move toward more personalized healthcare approaches.

To the best of our knowledge, no other bibliometric analysis on oral microbiome has been done using data from the PubMed database and performed factorial analysis. Also, utilizing automated tools for article screening in our research effectively mitigated the risk of subjective bias and facilitated large-scale data analysis. However, certain limitations must be acknowledged.

Limitations of this Study

A primary constraint of the study lies in the exclusive consideration of a single database for analysis. Additionally, the inability to conduct citation analysis is a noteworthy limitation, as the PubMed database does not support this type of examination. The sheer volume of articles in the bibliometric analysis renders it impractical to scrutinize each paper individually for specific data, necessitating reliance on systematic reviews for in-depth analysis. Also, owing to a constraint within the PubMed database, only the latest 10,000 articles were exportable in a text file.

**Figure 13:**
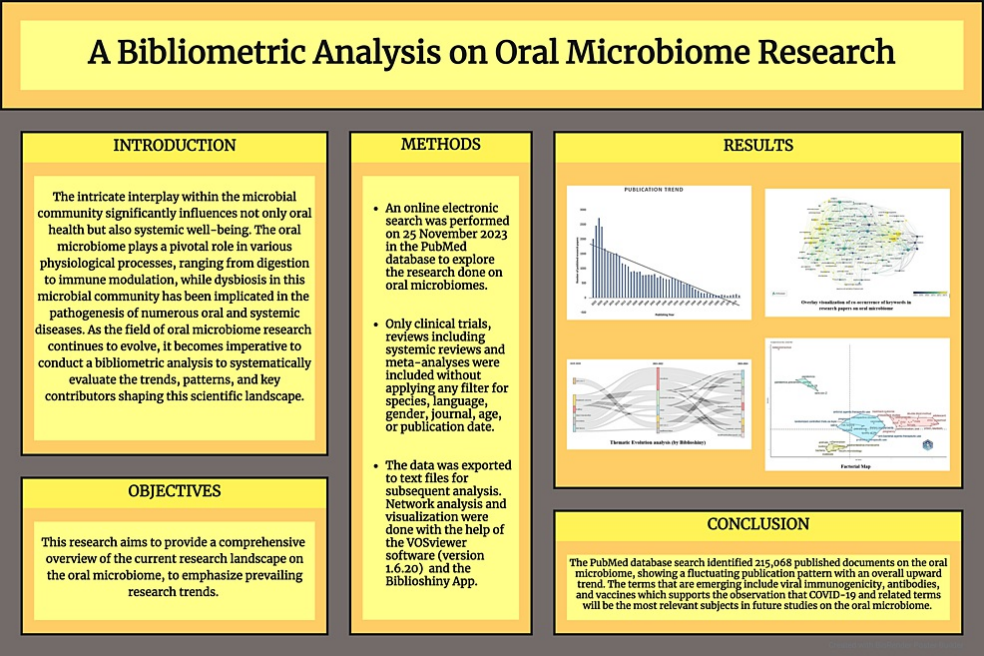
Illustrating the Principal findings of this paper. Notes: Image Credit: Susmita Sinha

Consequently, the analysis concentrated on this specific subset. Nonetheless, this study offers valuable insights into identifying research patterns, trends, and prominent sources and organizations beneficial for emerging researchers. The findings of this paper are depicted in Figure 13.

## Conclusions

The PubMed database search identified 215,068 published documents on the oral microbiome, showing a fluctuating publication pattern with an upward trend. Sichuan University and the International Journal of Molecular Sciences were recognized as the most productive organizations and relevant sources, respectively. A bibliometric analysis of the 10,000 most recent documents highlighted 23,545 keywords, indicating a focus on COVID-19 and the SARS-CoV-2 virus, particularly in adult females. The emerging terms include viral immunogenicity, antibodies, and vaccines, which supports the observation that COVID-19 and related terms will be the most relevant subjects in future studies on the oral microbiome. Factorial analysis has outlined the relationships between various research topics and subtopics within this field.

The thematic analysis highlights a sustained focus on SARS-CoV-2, treatment outcomes, microbiota, biofilms, and HIV over the last five years. Thus, the bibliometric analysis provides an overview of the most recent literature on oral microbiome research.
